# Investigation of founder effects for the Thr377Met *Myocilin* mutation in glaucoma families from differing ethnic backgrounds

**Published:** 2007-03-28

**Authors:** Alex W. Hewitt, John R. Samples, R. Rand Allingham, Irma Järvelä, George Kitsos, Subbaiah R. Krishnadas, Julia E. Richards, Paul R. Lichter, Michael B. Petersen, Periasamy Sundaresan, Janey L. Wiggs, David A. Mackey, Mary K. Wirtz

**Affiliations:** 1 Department of Ophthalmology, Flinders University, Adelaide, Australia; 2 Department of Ophthalmology, Casey Eye Institute-OHSU, Portland, OR; 3 Department of Ophthalmology, Duke University Eye Center, Durham, NC; 4 Department of Ophthalmology, University of Helsinki, Helsinki, Finland; 5 Department of Ophthalmology, University of Ioannina, Ioannina, Greece; 6 Department of Genetics, Aravind Medical Research Foundation, Madurai, India; 7 Department of Ophthalmology and Visual Sciences, University of Michigan, Ann Arbor, MI; 8 Department of Genetics, Institute of Child Health, Athens, Greece; 9 Department of Ophthalmology, Harvard Med School; Mass Eye & Ear Infirmary, Boston, MA; 10 Center for Eye Research Australia, University of Melbourne, Royal Victorian Eye and Ear Hospital, Melbourne, Australia

## Abstract

**Purpose:**

The aim of this study was to determine if there is a common founder for the Thr377Met *myocilin* mutation in primary open angle glaucoma (POAG) families with various ethnic backgrounds.

**Methods:**

Genomic DNA of 24 POAG-affected individuals from nine pedigrees with the Thr377Met mutation and 104 unaffected family members was genotyped with six microsatellite markers and four single nucleotide polymorphisms. The families were from Greece, India, Finland, the USA, and Australia. To assess the degree of linkage disequilibrium across *MYOC* in the general population we also investigated data generated from the HapMap consortium.

**Results:**

Three distinct haplotypes associated with the Thr377Met *myocilin* mutation were identified. The families from the USA and Greece, as well as the three Australian families originating from Greece and the former Yugoslavian Republic of Macedonia had one common haplotype. Interestingly, however, HapMap data suggest that linkage disequilibrium across *MYOC* was not strong.

**Conclusions:**

The Thr377Met myocilin mutation has arisen at least three separate times. Evidence for genetic founder effects in this prevalent age-related, yet heterogeneous, disease has important implications for future gene identification strategies.

## Introduction

Primary open angle glaucoma (POAG) is a complex heterogeneous disease and by the year 2020 is predicted to affect more than 50 million people worldwide [1]. Excavation of the optic disc with corresponding loss of visual field is the principal hallmark of POAG. Since its implication in the pathogenesis of POAG in 1997, numerous mutations in the *myocilin* (*MYOC*) gene have been identified and their specific phenotypes characterized [2,3]. The work recently performed by Shepard and colleagues has revealed that disease severity in *MYOC*-related POAG is influenced by exposure of a cryptic signaling site [4].

Worldwide, the Thr377Met *MYOC* mutation is one of the most commonly identified POAG-causing mutations and it has been previously identified in populations from five different continents. This specific mutation has been identified in four Australian-based families, two families residing in the United States of America, and one each from Greece, the former Yugoslavian Republic of Macedonia (FYROM), India, Finland, and Morocco [5-12]. The Thr377Met *MYOC* mutation is located within the COOH-terminal coding region and renders the protein insoluble [13]. Although this highly penetrant mutation appears to be sufficient to cause disease, evidence supporting gene-gene interaction in adult onset glaucoma has been described in a family harboring this variant [11]. Members of a Greek family who carried both the Thr377Met *MYOC* mutation and a haplotype associated with the GLC1C locus displayed greater disease severity than did those family members with only one disease variant [11].

In general, the Thr377Met mutation confers disease of intermediate severity, with patients typically being diagnosed in their fourth decade (weighted mean of age at diagnosis across described pedigrees=41.4 years). This is younger than the age at which patients with the Gln368Stop mutation are usually diagnosed, yet somewhat older than those carrying other *MYOC* mutations such as Pro370Leu, which is generally diagnosed around 15 years of age [5-12]. Following a similar pattern, patients with the Thr377Met mutation tend to have a maximum recorded intraocular pressure around 30 mmHg, lower than that described for mutations causing juvenile onset glaucoma (such as Pro370Leu), though generally higher than for the Gln368Stop mutation [7].

Inherited diseases with a relatively late onset may be more likely to have a common disease founder than those which manifest at a younger age. Evidence for a founding effect has previously been reported for the common Gln368Stop *MYOC* mutation; however, others, such as the Gly367Arg mutation, have arisen independently several times [14-16]. Small, local founder effects have also been identified for other less common mutations [9,14,17-19]. It is unclear whether a POAG variant causing intermediate disease severity would disseminate worldwide. Herein, we identified the haplotypes associated with the Thr377Met MYOC mutation to evaluate a possible founder effect in POAG families from various different ethnic backgrounds. To assess the degree of linkage disequilibrium across *MYOC* in the general population we also investigated data generated from the HapMap consortium.

## Methods

Nine Thr377Met *MYOC* glaucoma pedigrees from the United States of America, Greece, Finland, India, and Australia were recruited. The four Australian families had emigrated from Great Britain, Greece, and Macedonia. Both families from the United States of America were of Greek ethnicity (Table 1). The Indian proband lived in a Sino-Tibetan linguistic region. This study adhered to the Declaration of Helsinki and ethical approval had previously been obtained by each research group's respective local Institutional Review Board, as previously reported [5-12].

**Table 1:** Summary of phenotypic features of glaucomatous individuals carrying the Thr377Met *MYOC* mutation.

**Family designation**	**Country of residence (Ancestral Ethnicity)**	**n ofPOAG orOHT individuals described**	**Mean ± SE age at diagnosis (years)**	**Mean ± SE maximum recorded IOP (mmHg)**		**Previous Description of Pedigree**	
Vicl	Australia (British)	19	39 ±2.5	29.9= 1.8	[5]	
Vicll8	Australia (Greek)	1	57	60	[5]	
Vic119	Australia (Greek)	2	42 ±10	38±8	[5]	
Vic120	Australia (Macedonian)	1	47	30	[5]	
Epl	Greece (Greek)	9	51.3 ±4.9	32 ±3.8	[11]	
UMJG7	USA (Greek)	3	38 ± 1.7	44 ±1.7	[7]	
HMS7	USA (Greek)	1	42	24	[6]	
Finl	Finland (Finnish)	9	34.3 ± 8.8	33.2 ±5.5	[10]	
Indl	India (Sino-Tibetan)	1	52	44	[8]	
-	Morocco (Berber)	1	36	70	191	

In the table, abbreviations are: n, number; SE, standard error; POAG, primary open angle glaucoma; OHT, ocular hypertension; IOP, intraocular pressure; USA, United States of America.

Six microsatellite markers (D1S2851, MY3, My5, D1S1619, D1S2790, and D1S242) and four single nucleotide polymorphisms (rs3768570, rs235858, rs171000, and rs235873) located within a four Mb region surrounding the *MYOC* gene were genotyped in available subjects from each pedigree. Primer sequence and PCR conditions are available upon request. Haplotypes in each family were constructed by visual inspection of the genotype data, with no recombinations assumed between the markers. Genomic DNA was available from only one individual from the Indian cohort.

To estimate the probability of finding non-coincidental haplotypes segregating with the founded Thr377Met *MYOC* mutation, we determined the frequencies of the associated neighboring alleles in a sample of POAG patients originating from the same ethnic background. Thirty-three genealogically unrelated POAG patients of Greek ethnicity who, on direct sequencing, were found not to have the Thr377Met *MYOC* mutation, were recruited. The frequencies of the founded haplotype between the Greek Thr377Met *MYOC* mutation carrying patients and the Greek non-*MYOC* POAG patients was compared using Fisher's exact test (Intercooled Stata 7.0 for Windows; Stata Corporation, College Station, TX).

Allele frequencies and linkage disequilibrium across the chromosome region 1q24 was assessed using HapMap data [20]. Phase I and II data were accessed from public release 21. This data release contains all processed data from the project including genotypes from the Affymetric 500 k genotyping array. HapMap comprises data of genotyped individuals from the Centre d'Etude du Polymorphisme Humain collection in Utah; the Yoruba in Ibadan, Nigeria; the Han Chinese in Beijing, China; and the Japanese in Tokyo, Japan [20]. Haplotype blocks were reviewed using Haploview 3.32 [21] and defined as having an upper confidence interval maximum for strong recombination of 0.9 [22]. Markers with a minor allele frequency below 0.05 were excluded [22].

## Results

Genotyping of the microsatellite markers revealed a shared haplotype in mutation carriers across six families from Greece, the United States of America, and Australia (Table 2). All of these families were known to be of Greek or Macedonian ethnicity and recombination was found to have occurred in a region telomeric to *MYOC* between D1S1619 and D1S2790.

**Table 2:** Ancestral disease haplotype shared by individuals carrying the Thr377Met *MYOC* mutation from different ethnic backgrounds.

**Marker**	**Chromosome 1 location (Mb)**	**Greek**	**Australian (British)**	**Indian**	**Finnish**
D1S2851	167.048	187	187	177 181	187
rs3768570	167.89	-	G	G/A	A
rs235858	168.328	-	G	G/A	A
rs171000	168.33	-	G	G/T	1
MY3	168.336	176	174	174/ 178	178
rs235873	168.344	-	G	G/A	A
My5 (NGA17)	168.454	239	239	239	243
D1S1619	168.469	198	192	198/200	192
D1S2790	169.756	-	243	243 / 249	249
D1S242	171.104	-	215	221	217
Number of contributing pedigrees:		6	1	1	1
Number of mutation carrying subjects haplotyped:		26	12	1	4

To investigate whether the haplotype observed in the Greek/FYROM Thr377Met *MYOC* families was inherited only by chance we genotyped a small cohort of POAG patients of Greek ethnicity who were known not to have the Thr377Met *MYOC* mutation. Although the precise phase could not be fully determined because of the limited availability of other family members, one Greek patient with POAG was found to have markers (between My3 and D1S1619) in common with the founded Greek Thr377Met *MYOC* mutation. However, even with the conservative assumption that this patient did have the founded MYOC haplotype, our data supports the rejection of the hypothesis that the Greek Thr377Met haplotype merely represents frequent alleles (p<0.0001).

The families from Finland and Great Britain were found to have differing haplotypes from each other and from those which originated from Greece or Macedonia. This suggests that the Thr377Met *MYOC* mutation has arisen at least three times worldwide. The Indian proband was found to have some markers in common both with the Finnish pedigree and the family which originated from Great Britain. However, given that only one case from this family could be ascertained, the haplotype segregating with the Thr377Met *MYOC* mutation could not be fully determined.

**Figure 1:**
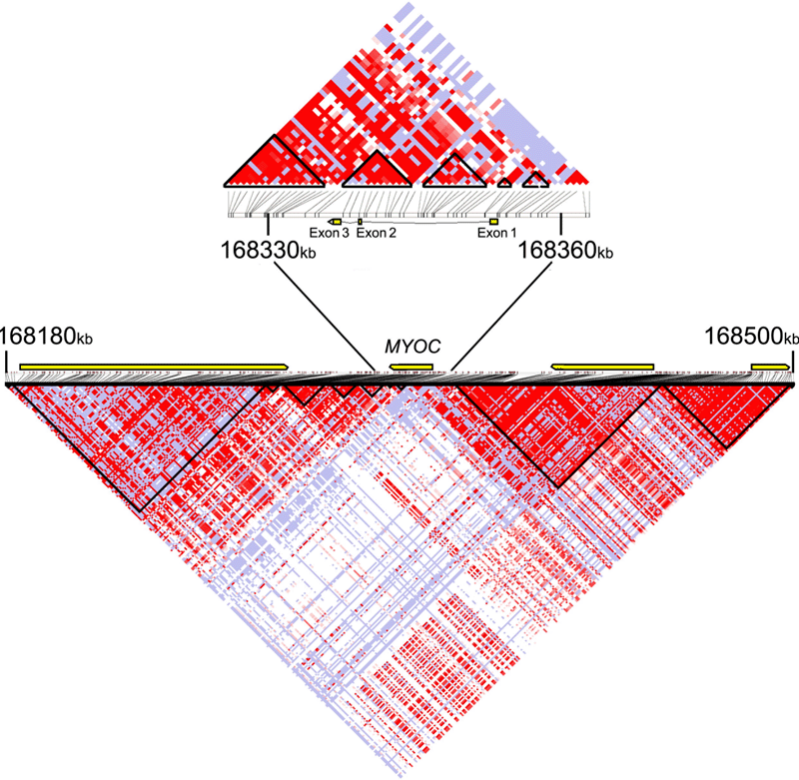
Haplotype blocks of the 320 kbp region containing the *myocilin* (*MYOC*) gene, in subjects from the Centre d'Etude du Polymorphisme Humain collection in Utah. The plotted D' statistic is orientated to the *MYOC* ideogram position and the strength of linkage disequilibrium is displayed in increasing shades of red for higher values. The relative gene locations are indicated by yellow bars.

Reviewing the data generated by the HapMap consortium revealed that linkage disequilibrium across *MYOC*, particularly in the Utah residents, was not strong (Figure 1). Larger linkage disequilibrium blocks over the genes neighboring *MYOC* (*HbxAg transactivated protein 2* and *vesicle-associated membrane protein 4*) were identified. In each population linkage disequilibrium was generally stronger across exons 2 and 3 compared to the 5' region of the *MYOC* gene.

## Discussion

Pre-symptomatic screening for glaucoma is an attractive prospect and although it may be currently uneconomical to introduce *MYOC* gene screening in a population-wide approach, targeted screening is warranted in selected populations. Our results imply that the Thr377Met *MYOC* mutation should be considered in glaucoma patients of Greek descent in particular.

**Figure 2:**
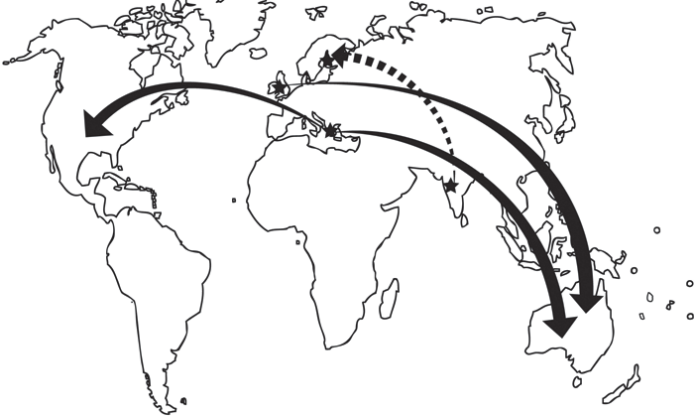
The worldwide spread of the Thr377Met *MYOC* mutation. Stars indicate location of known origin, solid arrows indicate known migrations, and the dashed arrow indicates an inferred migration.

A common haplotype was identified with the Thr377Met *MYOC* in six of the nine genealogically independent pedigrees studied. The northern boundary of Epirus, Greece, where the *Ep1* family in this report lives, forms the southern edge of the FYROM. Given this geographical proximity, we surmised that the Australian families from Greece and the FYROM have a common founder. However, the British, Finnish, and Indian families have a distinct haplotype from the Greek one, suggesting that the Thr377Met mutation has occurred de novo more than once (Figure 2). Unfortunately refinement of the haplotype associated with this mutation in the Indian case was limited by the lack of genetically informative relatives. Nonetheless, we hypothesize that a Finnish-Indian connection would be at least 1,500 years old, from when the Finno-Ugric (Huns) people migrated from Central Asia. To investigate this inferred dissemination further it would be imperative to collect additional samples from the Indian Thr377Met *MYOC* family, as well as other unrelated Indian POAG cases. A limitation of our work is that the location of the centromeric and telomeric recombination in each pedigree was not identified. Such information would allow for the identification of the minimal common genomic distance between mutation carriers and, in turn, provide an estimation of the age at which the Thr377Met mutation occurred, yet is beyond the scope of this work [23,24].

The population spread of human disease is significantly influenced by relative selection pressures and the existence of founder effects raises questions about whether such pressures are playing a role in *MYOC* allele frequencies. Our results, coupled with the late age at diagnosis (with the weighted mean across published pedigrees being 41.4 years), suggest that the Thr377Met *MYOC* mutation alone may not be significantly detrimental to reproduction. Although it is possible to arrive at a founder effect through non-selective processes such as genetic drift, it is plausible that this glaucoma-causing mutation may act as a protective factor against a separate ailment, thereby positively affecting biological fitness. Further insights may come from studies of Thr377Met allele frequencies in the Greek glaucoma populations. Evidence for genetic founder effects in this prevalent age-related, yet heterogeneous, disease has important implications for future gene identification strategies.

The haplotype structure across the comparatively small region surrounding *MYOC*, in conjunction with relatively small proportion of disease accounted for by mutations in *MYOC*, suggests that a high density SNP platform would be required for it to have been identified using a genome-wide association case-control approach. Nevertheless, genes with a different allelic architecture could be identified using such technology and, interestingly, a common disease haplotype has recently been associated with the common Y402H variant of *Complement Factor H* implicated in age-related macular degeneration [25]. Although confined conclusions based on the HapMap data must be approached with caution and the populations from which the data were generated should not be over-generalized, we found that linkage disequilibrium was marginally stronger across the regions with greater cross-species homology (particularly exon 3). Interestingly this supports other findings that on a genome-wide level sequence conservation appears not to be an important predictor of linkage disequilibrium [26].

In summary, the Thr377Met *MYOC* mutation has arisen at least three times in independent populations. Interestingly however, HapMap data suggest that linkage disequilibrium across *MYOC* is not strong. Evidence for genetic founder effects in this prevalent age-related, yet heterogeneous disease has important implications for future gene identification strategies.

## References

[1] Quigley HA, Broman AT (2006). The number of people with glaucoma worldwide in 2010 and 2020.. Br J Ophthalmol.

[2] Stone EM, Fingert JH, Alward WL, Nguyen TD, Polansky JR, Sunden SL, Nishimura D, Clark AF, Nystuen A, Nichols BE, Mackey DA, Ritch R, Kalenak JW, Craven ER, Sheffield VC (1997). Identification of a gene that causes primary open angle glaucoma.. Science.

[3] Hewitt AW, Craig JE, Mackey DA (2006). Complex genetics of complex traits: the case of primary open-angle glaucoma.. Clin Experiment Ophthalmol.

[4] Shepard AR Jacobson N Millar JC Pang IH Steely HT Searby CC Sheffield VC Stone EM Clark AF Glaucoma-causing myocilin mutants require the Peroxisomal Targeting Signal-1 Receptor (PTS1R) to elevate intraocular pressure. Hum Mol Genet 2007[Epub ahead of print] 17317787 10.1093/hmg/ddm001

[5] Mackey DA, Healey DL, Fingert JH, Coote MA, Wong TL, Wilkinson CH, McCartney PJ, Rait JL, de Graaf AP, Stone EM, Craig JE (2003). Glaucoma phenotype in pedigrees with the myocilin Thr377Met mutation.. Arch Ophthalmol.

[6] Wiggs JL, Allingham RR, Vollrath D, Jones KH, De La Paz M, Kern J, Patterson K, Babb VL, Del Bono EA, Broomer BW, Pericak-Vance MA, Haines JL (1998). Prevalence of mutations in TIGR/Myocilin in patients with adult and juvenile primary open-angle glaucoma.. Am J Hum Genet.

[7] Shimizu S, Lichter PR, Johnson AT, Zhou Z, Higashi M, Gottfredsdottir M, Othman M, Moroi SE, Rozsa FW, Schertzer RM, Clarke MS, Schwartz AL, Downs CA, Vollrath D, Richards JE (2000). Age-dependent prevalence of mutations at the GLC1A locus in primary open-angle glaucoma.. Am J Ophthalmol.

[8] Kanagavalli J, Krishnadas SR, Pandaranayaka E, Krishnaswamy S, Sundaresan P (2003). Evaluation and understanding of myocilin mutations in Indian primary open angle glaucoma patients.. Mol Vis.

[9] Melki R, Idhajji A, Driouiche S, Hassani M, Boukabboucha A, Akhayat O, Garchon H, Belmouden A (2003). Mutational analysis of the Myocilin gene in patients with primary open-angle glaucoma in Morocco.. Ophthalmic Genet.

[10] Puska P, Lemmela S, Kristo P, Sankila EM, Jarvela I (2005). Penetrance and phenotype of the Thr377Met Myocilin mutation in a large Finnish family with juvenile- and adult-onset primary open-angle glaucoma.. Ophthalmic Genet.

[11] Petersen MB, Kitsos G, Samples JR, Gaudette ND, Economou-Petersen E, Sykes R, Rust K, Grigoriadou M, Aperis G, Choi D, Psilas K, Craig JE, Kramer PL, Mackey DA, Wirtz MK (2006). A large GLC1C Greek family with a myocilin T377M mutation: inheritance and phenotypic variability.. Invest Ophthalmol Vis Sci.

[12] Allingham RR, Wiggs JL, De La Paz MA, Vollrath D, Tallett DA, Broomer B, Jones KH, Del Bono EA, Kern J, Patterson K, Haines JL, Pericak-Vance MA (1998). Gln368STOP myocilin mutation in families with late-onset primary open-angle glaucoma.. Invest Ophthalmol Vis Sci.

[13] Zhou Z, Vollrath D (1999). A cellular assay distinguishes normal and mutant TIGR/myocilin protein.. Hum Mol Genet.

[14] Faucher M, Anctil JL, Rodrigue MA, Duchesne A, Bergeron D, Blondeau P, Cote G, Dubois S, Bergeron J, Arseneault R, Morissette J, Raymond V, Quebec Glaucoma Network. (2002). Founder TIGR/myocilin mutations for glaucoma in the Quebec population.. Hum Mol Genet.

[15] Baird PN, Richardson AJ, Mackey DA, Craig JE, Faucher M, Raymond V (2005). A common disease haplotype for the Q368STOP mutation of the myocilin gene in Australian and Canadian glaucoma families.. Am J Ophthalmol.

[16] Hewitt AW, Bennett SL, Dimasi DP, Craig JE, Mackey DA (2006). A myocilin Gln368STOP homozygote does not exhibit a more severe glaucoma phenotype than heterozygous cases.. Am J Ophthalmol.

[17] Brezin AP, Adam MF, Belmouden A, Lureau MA, Chaventre A, Copin B, Gomez L, De Dinechin SD, Berkani M, Valtot F, Rouland JF, Dascotte JC, Bach JF, Garchon HJ (1998). Founder effect in GLC1A-linked familial open-angle glaucoma in Northern France.. Am J Med Genet.

[18] Angius A, De Gioia E, Loi A, Fossarello M, Sole G, Orzalesi N, Grignolo F, Cao A, Pirastu M (1998). A novel mutation in the GLC1A gene causes juvenile open-angle glaucoma in 4 families from the Italian region of Puglia.. Arch Ophthalmol.

[19] Hewitt AW, Bennett SL, Richards JE, Dimasi DP, Booth AP, Inglehearn C, Anwar R, Yamamoto T, Fingert JH, Heon E, Craig JE, Mackey DA (2007). Myocilin Gly252Arg mutation and glaucoma of intermediate severity in Caucasian individuals.. Arch Ophthalmol.

[20] The International HapMap Consortium (2003). The International HapMap Project.. Nature.

[21] Barrett JC, Fry B, Maller J, Daly MJ (2005). Haploview: analysis and visualization of LD and haplotype maps.. Bioinformatics.

[22] Gabriel SB, Schaffner SF, Nguyen H, Moore JM, Roy J, Blumenstiel B, Higgins J, DeFelice M, Lochner A, Faggart M, Liu-Cordero SN, Rotimi C, Adeyemo A, Cooper R, Ward R, Lander ES, Daly MJ, Altshuler D (2002). The structure of haplotype blocks in the human genome.. Science.

[23] Slatkin M (2000). Allele age and a test for selection on rare alleles.. Philos Trans R Soc Lond B Biol Sci.

[24] Genin E, Tullio-Pelet A, Begeot F, Lyonnet S, Abel L (2004). Estimating the age of rare disease mutations: the example of Triple-A syndrome.. J Med Genet.

[25] Hageman GS, Anderson DH, Johnson LV, Hancox LS, Taiber AJ, Hardisty LI, Hageman JL, Stockman HA, Borchardt JD, Gehrs KM, Smith RJ, Silvestri G, Russell SR, Klaver CC, Barbazetto I, Chang S, Yannuzzi LA, Barile GR, Merriam JC, Smith RT, Olsh AK, Bergeron J, Zernant J, Merriam JE, Gold B, Dean M, Allikmets R (2005). A common haplotype in the complement regulatory gene factor H (HF1/CFH) predisposes individuals to age-related macular degeneration.. Proc Natl Acad Sci USA.

[26] Kato M, Sekine A, Ohnishi Y, Johnson TA, Tanaka T, Nakamura Y, Tsunoda T (2006). Linkage disequilibrium of evolutionarily conserved regions in the human genome.. BMC Genomics.

